# Supplementary material for: Enhanced storage capacity with errors in scale-free Hopfield neural networks: An analytical study
Source: PLoS One. 2017 Oct 27;12(10):e0184683. doi: 10.1371/journal.pone.0184683 (PMC5659639; doi:10.1371/journal.pone.0184683)
Supplement: S1 File — This supporting information contains the detailed calculations of the free energy and order parameters using the replica analysis. (PDF) [file pone.0184683.s001.pdf]

Supporting information for "Enhanced storage capacity with errors in scale-free Hopfield neural networks: an analytical study"

Do-Hyun Kim[1*], Jinha Park[2], Byungnam Kahng[2*]

[1] Department of Physics, Sogang University, Seoul 04107, Korea
[2] CCSS, CTP and Department of Physics and Astronomy, Seoul National University, Seoul 08826, Korea

[*] Corresponding authors
E-mail: dohyunkim@sogang.ac.kr(D-HK) or bkahng@snu.ac.kr(BK)

This is the supporting information for the paper entitled **"Enhanced storage capacity with errors in scale-free Hopfield neural networks: an analytical study"**. This Supporting Information contains the detailed calculations of the free energy and order parameters using the replica analysis.

1 Hopfield model simulation on a random network

In order to give an insight for the Hopfield model simulation, we performed a simulation following the random updating method of the Hopfield model on a random network: An excitation pattern of a random network is denoted by $\{\xi_i^\mu\}$, where i is the index of the neuron, $\mu(=1,\ldots,p)$ is the index of the excitation pattern, and p is the number of existing patterns, equivalent to the number of stored memories. The value of ξ_i^μ is either $+1$ or -1. An initial spin configuration of each neural network is set to be the first pattern, i.e., $S_i(t=1)=\xi_i^{\mu=1}$ for each node i. Once this spin configuration is set up, at the next step $t=2$, a neuron i is randomly chosen, its Ising spin state is updated asynchronously as

$$S_i(t+1)=\mathrm{sgn}\Big(\sum_{j\in \mathrm{n.n.of}\, i} J_{ij}S_j(t)\Big), \qquad (1)$$

where the synapse efficacy J_{ij} is taken as $J_{ij}=(1/K)\sum_{\mu=1}^{p}\xi_i^\mu\xi_j^\mu$ following the Hebb's rule, and $K(\equiv 2L/N)$ is the mean degree of a given random network. If $\sum_j J_{ij}S_j(t)$ becomes zero, then $S_i(t+1)$ takes $+1$ definitely. This updating is repeated until the system reaches a stable fixed point, which is supposed to be the energy minimum state of the system. Thus the retrieved pattern is the spin configuration in the energy minimum state. This whole procedure completes one run.

An example of the Hopfield dynamics with a random network of size $N=9$ and the

number of edges $L = 15$ is shown in S1 Fig. Its adjacency matrix $\hat{A}$ is given as

$$\hat{A} = \begin{pmatrix} 0 & 1 & 1 & 0 & 1 & 1 & 0 & 0 & 0 \\ 1 & 0 & 1 & 1 & 1 & 0 & 1 & 0 & 1 \\ 1 & 1 & 0 & 1 & 0 & 0 & 1 & 1 & 0 \\ 0 & 1 & 1 & 0 & 0 & 0 & 0 & 0 & 0 \\ 1 & 1 & 0 & 0 & 0 & 1 & 0 & 1 & 0 \\ 1 & 0 & 0 & 0 & 1 & 0 & 0 & 0 & 0 \\ 0 & 1 & 1 & 0 & 0 & 0 & 0 & 0 & 1 \\ 0 & 0 & 1 & 0 & 1 & 0 & 0 & 0 & 0 \\ 0 & 1 & 0 & 0 & 0 & 0 & 1 & 0 & 0 \end{pmatrix}. \tag{2}$$

The two patterns $\xi^1 = \{1, -1, 1, 1, 1, 1, 1, -1, 1\} =$ "H" and $\xi^2 = \{-1, 1, -1, -1, 1, -1, -1, 1, -1\} =$ "I" are encoded to the intercoupling strengths J, following the Hebbian rule, Eq. (2) of the main paper.

$$J = \frac{1}{K} \hat{A} \otimes \left((\xi^1)^T \xi^1 + (\xi^2)^T \xi^2 \right) \tag{3}$$

$$= \frac{3}{5} \hat{A} \otimes \begin{pmatrix} 1 & -1 & 1 & 1 & 1 & 1 & 1 & -1 & 1 \\ -1 & 1 & -1 & -1 & -1 & -1 & -1 & 1 & -1 \\ 1 & -1 & 1 & 1 & 1 & 1 & 1 & -1 & 1 \\ 1 & -1 & 1 & 1 & 1 & 1 & 1 & -1 & 1 \\ 1 & -1 & 1 & 1 & 1 & 1 & 1 & -1 & 1 \\ 1 & -1 & 1 & 1 & 1 & 1 & 1 & -1 & 1 \\ 1 & -1 & 1 & 1 & 1 & 1 & 1 & -1 & 1 \\ -1 & 1 & -1 & -1 & -1 & -1 & -1 & 1 & -1 \\ 1 & -1 & 1 & 1 & 1 & 1 & 1 & -1 & 1 \end{pmatrix}$$

$$+ \frac{3}{5} \hat{A} \otimes \begin{pmatrix} 1 & -1 & 1 & 1 & -1 & 1 & 1 & -1 & 1 \\ -1 & 1 & -1 & -1 & 1 & -1 & -1 & 1 & -1 \\ 1 & -1 & 1 & 1 & -1 & 1 & 1 & -1 & 1 \\ 1 & -1 & 1 & 1 & -1 & 1 & 1 & -1 & 1 \\ -1 & 1 & -1 & -1 & 1 & -1 & -1 & 1 & -1 \\ 1 & -1 & 1 & 1 & -1 & 1 & 1 & -1 & 1 \\ 1 & -1 & 1 & 1 & -1 & 1 & 1 & -1 & 1 \\ -1 & 1 & -1 & -1 & 1 & -1 & -1 & 1 & -1 \\ 1 & -1 & 1 & 1 & -1 & 1 & 1 & -1 & 1 \end{pmatrix} \tag{4}$$

$$= \begin{pmatrix} 0 & -\frac{6}{5} & \frac{6}{5} & \frac{6}{5} & 0 & 0 & 0 & 0 & 0 \\ -\frac{6}{5} & 0 & -\frac{6}{5} & 0 & 0 & 0 & -\frac{6}{5} & \frac{6}{5} & 0 \\ \frac{6}{5} & -\frac{6}{5} & 0 & \frac{6}{5} & 0 & \frac{6}{5} & 0 & -\frac{6}{5} & 0 \\ \frac{6}{5} & 0 & \frac{6}{5} & 0 & 0 & \frac{6}{5} & \frac{6}{5} & 0 & \frac{6}{5} \\ 0 & 0 & 0 & 0 & 0 & 0 & 0 & 0 & 0 \\ 0 & 0 & \frac{6}{5} & \frac{6}{5} & 0 & 0 & 0 & 0 & \frac{6}{5} \\ 0 & -\frac{6}{5} & 0 & \frac{6}{5} & 0 & 0 & 0 & 0 & 0 \\ 0 & \frac{6}{5} & -\frac{6}{5} & 0 & 0 & 0 & 0 & 0 & 0 \\ 0 & 0 & 0 & \frac{6}{5} & 0 & \frac{6}{5} & 0 & 0 & 0 \end{pmatrix}, \tag{5}$$

where $\otimes$ denotes an elementwise multiplication between two matrices and the mean degree $K = \frac{5}{3}$. Now starting from an initial condition $S(t=0) = \{1, -1, -1, 1, 1, -1, 1, -1, 1\}$, the system evolves under Eq. (1) and reaches a stationary state $\xi^1 =$ "H". Also from an initial condition

$S(t=0) = \{1, 1, -1, -1, 1, -1, -1, 1, 1\}$, the system evolves under Eq. (1) and reaches a stationary state $\xi^2 =$ "I". See S1 Fig (c) and (d).

2 Hebbian rule

1) The system's energy decreases in time.
(i) Suppose S_k was flipped at time t, $S_k(t+1) = -S_k(t)$ and $S_i(t+1) = S_i(t)$ for all other indices $i \neq k$. This implies,

$$\mathrm{sgn}\left(\sum_i J_{ik} S_i(t)\right) \neq \mathrm{sgn}\left(S_k(t)\right).$$

Then,

$$\begin{aligned} H(t+1) - H(t) &= -\sum_{ij} J_{ij} S_i(t+1) S_j(t+1) + \sum_{ij} J_{ij} S_i(t) S_j(t) \\ &= -2 \sum_{i \neq k} J_{ik} S_i(t+1) S_k(t+1) \\ &= 2 \left(\sum_{i \neq k} J_{ik} S_i(t)) \right) S_k(t) < 0 \end{aligned}$$

(ii) Suppose if S_k was not flipped, i.e. $\vec{S}(t+1) = \vec{S}(t)$. Thus $H(t+1) - H(t) = 0$.
Therefore with the asynchronous sgn updating rule, the energy of the system decreases monotonically. $H(t+1) - H(t) \leq 0$.

2) By the Hebbian encoding, each stored pattern corresponds to a local energy minimum.
Let $S = \xi^1 + \delta S$.

$$H[S] = -\sum_{ij} J_{ij} S_i S_j = -\sum_{ij} J_{ij} (\xi^1 + \delta S)_i (\xi^1 + \delta S)_j$$

$$\begin{aligned} \left.\frac{\delta H}{\delta S}\right|_{S=\xi^1} &= -2 \sum_{ij} J_{ij} \xi_i^1 = -\frac{2}{N} \sum_{i \neq j} \sum_{\mu=1}^{p} \xi_i^\mu \xi_j^\mu \xi_i^1 \\ &= -\sum_\mu \Big(\sum_j \xi_j^\mu \Big) \Big(\frac{1}{N} \sum_i \xi_i^\mu \xi_i^1 \Big) + \frac{2}{N} \sum_\mu \Big(\sum_i \xi_i^1 \Big) = 0. \end{aligned}$$

where $\sum_j \xi_j^\mu = 0$, because the stored patterns are assumed unbiased, and $\frac{1}{N} \sum_i \xi_i^\mu \xi_i^1 = 0$ because the stored patterns $\xi^\mu, \xi^\nu, \cdots$ are assumed uncorrelated.

$$\begin{aligned} \left.\frac{\delta^2 H}{\delta S^2}\right|_{S=\xi^1} &= -\sum_{ij} J_{ij} \\ &= -\frac{1}{N} \sum_{i \neq j} \sum_{\mu=1}^{p} \xi_i^\mu \xi_j^\mu \\ &= -\frac{1}{N} \sum_\mu \Big(\sum_i \xi_i^\mu \Big)^2 + \frac{1}{N} \sum_\mu \sum_i \left(\xi_i^\mu\right)^2 = p > 0. \end{aligned}$$

The argument also holds for $\xi^2, \xi^3, \cdots \xi^p$. Therefore each pattern ξ^μ is a local energy minimum. The Hebbian rule shapes the energy function to become quadratic near each pattern ξ^μ. For random networks uncorrelated with patterns, $\hat{A}_{ij}$ randomly sets some of the terms to zero and argument 2) is effectively unchanged.

From the above arguments 1) and 2), we conclude that the stored patterns ξ^μ are stationary states of the Hopfield dynamics. However, these states are not the only stationary states. Some other local energy minima can exist, which are states usually given as superpositions of the existing patterns. In the SG regime, the number of local energy minima become much larger than the number of stored patterns, hindering the memory retrieval process.

3 Solution of the Hopfield model on scale-free networks

Now we consider the Hopfield model on SF networks. Since Amit *et al.* successfully applied replica analysis of spin glass theory to the Hopfield model [1–3], neural networks have been regarded as analogous systems of spin glasses (SGs) [4, 5]. The SG transitions in Euclidean space have already been studied by means of various theoretical methods [4, 5]. Most of such studies have been carried out on regular lattices or for the infinite-range interaction model on fully connected graphs. To study the SG transitions on SF networks, we followed the previous approach used for the dilute Ising SG model with infinite-range interactions, i.e., the Ising SG model on the ER graph, first performed by Viana and Bray [6–11], and applied it successfully to the static model of SF networks [12]. The Ghatak-Sherrington SG model was also studied on the same networks [13].

In the spin glass and its analogous systems, the replica method is conventionally used to evaluate the free energy [4, 5] to calculate the ensemble averages in $-\beta F = \langle\langle \ln Z\rangle_\xi\rangle_K$, where Z is the partition function for a given distribution of $\{\xi_i\}$ and $\{K\}$ on a particular graph G and $\beta = 1/T$. Here, once ξ and K are given, they become fixed throughout all retrieval processes, thus they are regarded as quenched variables in statistical physics. For such systems with the quenched variables, we cannot calculate $\langle\langle \ln Z\rangle_\xi\rangle_K$ mathematically. Instead, we can carry out such calculations with the so-called replica method, i.e., the mathematical relation $X^n = e^{n\ln X} \approx 1 + n\ln X$ as $n \to 0$. Therefore, using the replica method, we obtain the following relation: $-\beta F = \langle\langle \ln Z\rangle_\xi\rangle_K = \lim_{n\to 0}[\langle\langle Z^n\rangle_\xi\rangle_K - 1]/n$. By evaluating the n-th power of the partition function $\langle\langle Z^n\rangle_\xi\rangle_K$, we finally obtain the free energy. For simplicity, only the first pattern ($\mu = 1$) is retrieved. This simplification is based on the fact that the replica symmetry only appears to be broken in a particular case at $T = 0$ [1].

Using the replica method, we evaluate the n-th power of the partition function Z^n,

$$\begin{aligned}\langle\langle Z^n\rangle_\xi\rangle_K &= \mathrm{Tr}\,\Big\langle\Big\langle \exp\Big(\frac{\beta}{K}\sum_{(ij)\in G}\sum_{\mu=1}^{p}\sum_{\alpha}^{n}\xi_i^\mu\xi_j^\mu S_i^\alpha S_j^\alpha\Big)\Big\rangle_\xi\Big\rangle_K \\ &= \mathrm{Tr}\,\exp\Big[\sum_{i<j}\ln\Big\{1 + f_{ij}\Big(\Big\langle\exp\Big(\frac{\beta}{K}\sum_{\mu=1}^{p}\sum_{\alpha}^{n}\xi_i^\mu\xi_j^\mu S_i^\alpha S_j^\alpha\Big)\Big\rangle_\xi - 1\Big)\Big\}\Big], \quad (6)\end{aligned}$$

where the trace Tr is taken over all replicated spins $S_i^\alpha(=\pm 1)$, and $\alpha = 1, \ldots, n$ is the

replica index. Using the relation,

$$\Big\langle \exp\Big(\frac{\beta}{K}\sum_{\mu=1}^{p}\sum_{\alpha}^{n}\xi_i^\mu\xi_j^\mu S_i^\alpha S_j^\alpha\Big)\Big\rangle_\xi = \Big\langle \prod_{\mu=1}^{p}\prod_{\alpha}^{n}\Big[\cosh(\beta/K)\Big(1+\xi_i^\mu\xi_j^\mu S_i^\alpha S_j^\alpha\tanh(\beta/K)\Big)\Big]\Big\rangle_\xi, \quad (7)$$

$\langle\langle Z^n\rangle_\xi\rangle_K$ term becomes

$$\begin{aligned}\langle\langle Z^n\rangle_\xi\rangle_K &\overset{n\to 0}{=} \mathrm{Tr}\prod_{i\neq j}\exp\Big[\frac{1}{2}\Big\langle\Big[NKw_iw_j + NK\mathbf{T}_1 w_i\xi_i^1 S_i^\alpha w_j\xi_j^1 S_j^\alpha+\cdots\Big]\times \\ &\prod_{\mu=2}^{p}\Big[1+\mathbf{T}_1\xi_i^\mu S_i^\alpha\xi_j^\mu S_j^\alpha+\cdots\Big]\Big\rangle_\xi - NKw_iw_j\Big] \\ &= \mathrm{Tr}\prod_{i\neq j}\exp\frac{1}{2}\Big\langle NK\mathbf{T}_1(w_i\xi_i^1 S_i^\alpha)(w_j\xi_j^1 S_j^\alpha)+\sum_{\mu=2}^{p}NK\mathbf{T}_1(w_i\xi_i^\mu S_i^\alpha)(w_j\xi_j^\mu S_j^\alpha) \\ &+NK\mathbf{T}_2(w_i\xi_i^1 S_i^\alpha\xi_i^1 S_i^\beta)(w_j\xi_j^1 S_j^\alpha\xi_j^1 S_j^\beta) \\ &+\sum_{\mu=2}^{p}NK\mathbf{T}_2(w_i\xi_i^\mu S_i^\alpha\xi_i^\mu S_i^\beta)(w_j\xi_j^\mu S_j^\alpha\xi_j^\mu S_j^\beta)+\cdots\Big\rangle_\xi, \quad (8)\end{aligned}$$

where

$$\begin{aligned}\mathbf{T}_l(T) &\equiv \cosh^n(\beta/K)\tanh^l(\beta/K)\overset{n\to 0}{\longrightarrow}\tanh^l(\beta/K) \\ &(l=1,2,\ldots). \quad (9)\end{aligned}$$

Because all p stored patterns were generally randomly, we can examine the retrieval pattern of the first pattern ($\mu=1$) only. Thus, we separate the contribution of the first pattern from the rest. The $\langle\langle Z^n\rangle_\xi\rangle_K$ term can be linearized using the Hubbard-Stratonovich transformation [14, 15]

$$\exp\{\frac{1}{2}\lambda a^2\} = \sqrt{\frac{\lambda}{2\pi}}\int_{-\infty}^{\infty}dx\ \exp\Big\{-\frac{1}{2}\lambda x^2+\lambda a x\Big\}\ . \quad (10)$$

Therefore,

$$\begin{aligned}\langle\langle Z^n\rangle_\xi\rangle_K &= \mathrm{Tr}\int\prod_{\mu}^{p}\prod_{\alpha}^{n}dm_\alpha^\mu\ \Big\langle\exp\Big[NK\mathbf{T}_1\sum_{\alpha}^{n}\Big\{-\frac{1}{2}(m_\alpha^1)^2+\sum_{i}^{N}m_\alpha^1(w_i\xi_i^1 S_i^\alpha)\Big\}\Big] \\ &\times\exp\Big[NK\mathbf{T}_1\sum_{\mu=2}^{p}\sum_{\alpha}^{n}\Big\{-\frac{1}{2}(m_\alpha^\mu)^2+\sum_{i}^{N}m_\alpha^\mu(w_i\xi_i^\mu S_i^\alpha)\Big\}\Big]\cdots\Big\rangle_\xi \\ &= \mathrm{Tr}\int\prod_{\mu}^{p}\prod_{\alpha}^{n}dm_\alpha^\mu\ \exp\Big[NK\mathbf{T}_1\sum_{\alpha}^{n}\Big\{-\frac{1}{2}(m_\alpha^1)^2+\sum_{i}^{N}m_\alpha^1(w_i\xi_i^1 S_i^\alpha)\Big\}\Big]\times \\ &\exp\Big[NK\mathbf{T}_1\sum_{\mu=2}^{p}\sum_{\alpha}^{n}\Big\{-\frac{1}{2}(m_\alpha^\mu)^2\Big\} \\ &+\sum_{\mu=2}^{p}\sum_{i}^{N}\ln\cosh\Big(NK\mathbf{T}_1\sum_{\alpha}^{n}m_\alpha^\mu(w_iS_i^\alpha)\Big)\Big]\cdots. \quad (11)\end{aligned}$$

In the thermodynamic limit ($N \to \infty$), we expand $\ln\cosh(\cdots)$ to the second order of m^μ_α, so that

$$\exp\Big[NK\mathbf{T}_1\sum_{\mu=2}^{p}\sum_{\alpha}^{n}\Big\{-\frac{1}{2}(m^\mu_\alpha)^2\Big\}+\sum_{\mu=2}^{p}\sum_{i}^{N}\ln\cosh\Big(NK\mathbf{T}_1\sum_{\alpha}^{n}m^\mu_\alpha(w_iS^\alpha_i)\Big)\Big]$$
$$\simeq \exp\Big[-\frac{1}{2}NK\mathbf{T}_1\sum_{\mu=2}^{p}\sum_{\alpha\beta}^{n}m^\mu_\alpha K_{\alpha\beta}m^\mu_\beta\Big], \qquad (12)$$

where $K_{\alpha\beta} \equiv \delta_{\alpha\beta} - K\mathbf{T}_1\sum_i^N w_iS^\alpha_iS^\beta_i$. Since Eq. (12) is quadratic in m^μ_α, the integral over m^μ_α can be carried out using of the multi-variable Gaussian integral as follows:

$$\int\prod_{\mu=2}^{p}dm^\mu_\alpha\ \exp\Big[-\frac{1}{2}NK\mathbf{T}_1\sum_{\mu=2}^{p}\sum_{\alpha\beta}^{n}m^\mu_\alpha K_{\alpha\beta}m^\mu_\beta\Big] \simeq (\det\mathbf{K})^{-(p-1)/2}$$
$$= \int\prod_{(\alpha\beta)}^{n}dq_{\alpha\beta}\ \delta\Big(q_{\alpha\beta}-\sum_{i}^{N}w_iS^\alpha_iS^\beta_i\Big)\exp\Big(-\frac{p-1}{2}\ \mathrm{Tr}_n\ln\{\mathbf{I}-K\mathbf{T}_1(\mathbf{I}+\mathbf{Q})\}\Big), \qquad (13)$$

where $(\alpha\beta)$ denotes a summation over α and $\beta(\neq\alpha)$. Here, we used the fact that the diagonal element $K_{\alpha\alpha}$ of the matrix $\mathbf{K}$ equals to $1-K\mathbf{T}_1$ and the off-diagonal element becomes

$$K_{\alpha\beta} = -K\mathbf{T}_1\sum_{i}^{N}w_iS^\alpha_iS^\beta_i \equiv -K\mathbf{T}_1q_{\alpha\beta}, \qquad (14)$$

which was expressed in terms of the spin glass order parameter $q_{\alpha\beta}$. The matrix $\mathbf{Q}$ consists of zeros along the diagonal elements and the off-diagonal elements $q_{\alpha\beta}$. Tr_n is the trace of the $n\times n$ matrix. Using the Fourier representation of the δ function in Eq. (13) with integral variable $\tilde{r}_{\alpha\beta}$, we can obtain the result:

$$\begin{aligned}\langle\langle Z^n\rangle_\xi\rangle_K \;=\; \mathrm{Tr}\int\prod_{\alpha}^{n}dm^1_\alpha\prod_{(\alpha\beta)}^{n}dq_{\alpha\beta}\prod_{(\alpha\beta)}^{n}d\tilde{r}_{\alpha\beta}\exp\Big[NK\mathbf{T}_1\sum_{\alpha}^{n}\Big\{-\frac{1}{2}(m^1_\alpha)^2+\\ \sum_{i}^{N}m^1_\alpha(w_i\xi^1_iS^\alpha_i)\Big\}\Big]\\ \times\exp\Big[\mathrm{i}NK\mathbf{T}_1\sum_{(\alpha\beta)}^{n}\tilde{r}_{\alpha\beta}\Big(q_{\alpha\beta}-\sum_{i}^{N}w_iS^\alpha_iS^\beta_i\Big)\Big]\\ \times\exp\Big(-\frac{p-1}{2}\ \mathrm{Tr}_n\ln\{(1-K\mathbf{T}_1)\mathbf{I}-K\mathbf{T}_1\mathbf{Q}\}\Big).\end{aligned} \qquad (15)$$

The many-spin coupled problem is transferred to an average over a decoupled problem of single spins. For this simplification, we deal with inter-replica couplings in the single-spin problem. So we have

$$\langle\langle Z^n\rangle_\xi\rangle_K = \mathrm{Tr}\int\prod_{\alpha}^{n}dm^1_\alpha\prod_{(\alpha\beta)}^{n}dq_{\alpha\beta}\prod_{(\alpha\beta)}^{n}d\tilde{r}_{\alpha\beta}\ \exp\{-NG(m^1_\alpha,q_{\alpha\beta},\tilde{r}_{\alpha\beta})\}, \qquad (16)$$

where

$$G(m_\alpha^1, q_{\alpha\beta}, \tilde{r}_{\alpha\beta}) \equiv \frac{1}{2}K\mathbf{T}_1 \sum_\alpha^n (m_\alpha^1)^2 - \mathrm{i}K\mathbf{T}_1 \sum_{(\alpha\beta)}^n \tilde{r}_{\alpha\beta} q_{\alpha\beta} + \frac{a}{2}\, \mathrm{Tr}_n \ln\{(1-K\mathbf{T}_1)\mathbf{I} - K\mathbf{T}_1\mathbf{Q}\} - \ln \mathrm{Tr}_i \exp(\tilde{\mathcal{H}}_i) \tag{17}$$

with

$$\tilde{\mathcal{H}}_i \equiv NK\mathbf{T}_1 \Big(\sum_\alpha^n m_\alpha^1 (w_i \xi_i^1 S_i^\alpha) - \mathrm{i} \sum_{(\alpha\beta)}^n \tilde{r}_{\alpha\beta}(w_i S_i^\alpha S_i^\beta) \Big). \tag{18}$$

Here we used $p - 1 \simeq p = aN$ and the trace Tr_i is now at a single spin site.

In the thermodynamic limit ($N \to \infty$) the integrals can be performed by the steepest descent method:

$$\int dy\ \exp\{-NG(y)\} \simeq \int dy\ \exp\Big\{ - NG(y_0) - \frac{1}{2}NG''(y_0)(y-y_0)^2 + \cdots \Big\}, \tag{19}$$

where $G'(y_0) = 0$ determines a saddle point y_0. The Gaussian term can be ignored for $N \to \infty$, provided that $G''(y_0) > 0$. Otherwise, the resulting integral diverges and the saddle point procedure fails. Assuming $G''(y) > 0$, we replace y with their stationary value. Therefore, we can define three order parameters as follows: i) m_α^μ represents the extent to which the μ-th pattern of memory ξ^μ and the α-th state of the system S^α overlap with each other;

$$m_\alpha^\mu = \sum_i^N w_i \langle \xi_i^\mu S_i^\alpha \rangle. \tag{20}$$

ii) $q_{\alpha\beta}$ is the spin glass order parameter representing the extent to which the two states α and β of the replica overlap each other;

$$q_{\alpha\beta} = \sum_i^N w_i \langle S_i^\alpha S_i^\beta \rangle. \tag{21}$$

iii) $r_{\alpha\beta}$ represents the extent to which the two different m_α^μ's overlap each other;

$$r_{\alpha\beta} \equiv \frac{N}{p} \sum_{\mu=2}^p m_\alpha^\mu m_\beta^\mu = \frac{1}{a} \sum_{\mu=2}^p q_{\alpha\beta} = -\frac{2\mathrm{i}}{aK\mathbf{T}_1} \tilde{r}_{\alpha\beta}. \tag{22}$$

So, $r_{\alpha\beta}$ can be understood as the sum of the effects of non-retrieved patterns. Here, the average is evaluated through $\langle A \rangle \equiv \mathrm{Tr}_i A \exp \tilde{\mathcal{H}}_i / \mathrm{Tr}_i \exp \tilde{\mathcal{H}}_i$.

Therefore, the free energy becomes

$$n\beta f = \frac{1}{2}K\mathbf{T}_1 \sum_\alpha^n (m_\alpha^1)^2 + \frac{1}{2}aK^2\mathbf{T}_2 \sum_{(\alpha\beta)}^n r_{\alpha\beta} q_{\alpha\beta} + \frac{a}{2}\, \mathrm{Tr}_n \ln\{(1-K\mathbf{T}_1)\mathbf{I} - K\mathbf{T}_1\mathbf{Q}\} - \ln \mathrm{Tr}_i \exp(\tilde{\mathcal{H}}_i), \tag{23}$$

where

$$\tilde{\mathcal{H}}_i = NK\mathbf{T}_1 \sum_\alpha^n m_\alpha^1 (w_i \xi_i^1 S_i^\alpha) + \frac{1}{2}aNK^2\mathbf{T}_2 \sum_{(\alpha\beta)}^n r_{\alpha\beta}(w_i S_i^\alpha S_i^\beta). \tag{24}$$

4 Replica-symmetric solutions

Here we take the replica-symmetric (RS) assumption to set $q_{\alpha\beta} = q$ and $r_{\alpha\beta} = r$ for all $\alpha \neq \beta$, and $m^1_\alpha = m$ for all α. The RS solution $(m,\, q,\, r)$ is the simplest one among several solutions obtained by the free energy (Eq. (23)). As we will be confirmed, the RS solution is stable for entire temperature regime but for very low temperature region near zero. Thus, using only the RS solution, we can analyze various characteristics of the Hopfield neural network.

Then the free energy is given by

$$\beta f = \lim_{n\to 0} \frac{1}{n}\left[\frac{1}{2}K\mathbf{T}_1 n m^2 + \frac{1}{2}K^2\mathbf{T}_2 n(n-1)arq + \frac{a}{2}\ \mathrm{Tr}_n \ln\{(1-K\mathbf{T}_1)\mathbf{I} - K\mathbf{T}_1\mathbf{Q}\} - \ln \mathrm{Tr}_i \exp(\tilde{\mathcal{H}}')\right] \tag{25}$$

with the effective Hamiltonian $\tilde{\mathcal{H}}'$

$$\tilde{\mathcal{H}}' \equiv NK\mathbf{T}_1 w_i m \xi^1 \sum_{\alpha}^{n} S^\alpha + \frac{1}{2}NK^2\mathbf{T}_2 w_i ar \sum_{(\alpha\beta)}^{n} S^\alpha S^\beta. \tag{26}$$

To calculate the trace of the third term of Eq. (25), it should be noted that the eigenvectors of the matrix $\mathbf{Q}$ are, first, the uniform one ${}^t(1,1,\cdots,1)$ and, second, the form of ${}^t(1,0,\cdots,0,-1,0,\cdots,0)$. So, the eigenvalue of the first eigenvector of the matrix $(1-K\mathbf{T}_1)\mathbf{I} - K\mathbf{T}_1\mathbf{Q}$ is $1-K\mathbf{T}_1-(n-1)K\mathbf{T}_1 q$ (no degeneracy), and the eigenvalue of the second eigenvector is $1-K\mathbf{T}_1+K\mathbf{T}_1 q$ (degeneracy $n-1$). Thus, in the limit $n \to 0$,

$$\begin{aligned}\frac{1}{n}\mathrm{Tr}_n \ln\{(1-K\mathbf{T}_1)\mathbf{I} - K\mathbf{T}_1\mathbf{Q}\} &= \frac{1}{n}\ln(1-K\mathbf{T}_1-(n-1)K\mathbf{T}_1 q) \\ &\quad + \frac{n-1}{n}\ln(1-K\mathbf{T}_1+K\mathbf{T}_1 q) \\ &\stackrel{n\to 0}{\longrightarrow} \ln(1-K\mathbf{T}_1+K\mathbf{T}_1 q) - \frac{K\mathbf{T}_1 q}{1-K\mathbf{T}_1+K\mathbf{T}_1 q}.\end{aligned} \tag{27}$$

When we put $\xi^1 = 1$, we finally obtain

$$\begin{aligned}\beta f &= \frac{1}{2}K^2\mathbf{T}_2 ar(1-q) + \frac{1}{2}K\mathbf{T}_1 m^2 + \frac{a}{2}\left[\ln(1-K\mathbf{T}_1+K\mathbf{T}_1 q) - \frac{K\mathbf{T}_1 q}{1-K\mathbf{T}_1+K\mathbf{T}_1 q}\right] \\ &\quad - \int \mathcal{D}z \frac{1}{N}\sum_{i=1}^{N} \ln\left[2\cosh \eta_i(z)\right].\end{aligned} \tag{28}$$

where $\int \mathcal{D}z\ \cdots \equiv \frac{1}{\sqrt{2\pi}}\int_{-\infty}^{\infty} dz\ \exp\{-\frac{1}{2}z^2\}\ \cdots$, and $\eta_i(z) \equiv K\mathbf{T}_1(z\sqrt{Nw_i ar} + Nw_i m)$.

We can determine m, r, and q by imposing the condition that f resumes the stable extrema when they are the RS solutions. From this extremal condition, we can obtain the self-consistent equations of m, q, and r as follows:

$$m = \int \mathcal{D}z \sum_{i=1}^{N} w_i \tanh \eta_i(z) \tag{29}$$

$$q = \int \mathcal{D}z \sum_{i=1}^{N} w_i \tanh^2 \eta_i(z) \tag{30}$$

$$r = \frac{q}{(1-K\mathbf{T}_1+K\mathbf{T}_1 q)^2}. \tag{31}$$

The Almeida-Thouless (AT) line, i.e., the condition satisfying $G''(y_0) = 0$ in Eq. (19), is simply given by [3,16]

$$(1 - K\mathbf{T}_1 + K\mathbf{T}_1 q)^2 - K^2\mathbf{T}_2 a \int \mathcal{D}z \sum_{i=1}^{N} N w_i^2 \ \mathrm{sech}^4 \eta_i(z) = 0. \tag{32}$$

The AT lines for various γ values locate in very low temperature region near zero so that even the SG and M phases become stable under replica symmetry. Note that the dotted black line near zero temperature in each panel of S2 Fig represents the AT line. Thus, the replica-symmetric solution is valid over almost the entire region.

We consider a particular case $T \to 0$ (i.e., $\mathbf{T}_l \to 1 \ \ (l = 1, 2)$). In this limit the "tanh" reduces to a step function

$$\tanh \eta_i(z) \to \mathrm{sgn}(\eta_i(z)), \tag{33}$$

where $\eta_i(z) = K(z\sqrt{Nw_i a r} + N w_i m)$. Then the equation for m becomes

$$\begin{aligned} m &= \int \mathcal{D}z \sum_{i=1}^{N} w_i \ \mathrm{sgn}(\eta_i(z)) \\ &= \sum_{i=1}^{N} w_i \frac{2}{\sqrt{2\pi}} \int_0^{\sqrt{\frac{Nw_i}{ar}}m} dz \ e^{-\frac{1}{2}z^2} \\ &= \sum_{i=1}^{N} w_i \ \mathrm{erf}\Big(\sqrt{\frac{Nw_i}{2ar}} m\Big), \end{aligned} \tag{34}$$

where $\mathrm{erf}(\cdots)$ means the error function. The parameter q approaches one, i.e., $q \to 1$ in the zero temperature limit. A simple equation in this limit is readily obtained from $\partial(\beta f)/\partial r = 0$:

$$\begin{aligned} NK(1-q) &= \sum_{i=1}^{N} \sqrt{\frac{Nw_i}{ar}} \int \mathcal{D}z \ z \ \mathrm{sgn}(\eta_i(z)) \\ &= \sum_{i=1}^{N} \sqrt{\frac{Nw_i}{ar}} \frac{2}{\sqrt{2\pi}} \int_{\sqrt{\frac{Nw_i}{ar}}m}^{\infty} dz \ z \ e^{-\frac{1}{2}z^2} \\ &= \sum_{i=1}^{N} \sqrt{\frac{2Nw_i}{\pi a r}} \ \exp\Big(-\frac{Nw_i}{2ar} m^2\Big). \end{aligned} \tag{35}$$

Therefore, at zero temperature, m, q and r are obtained as

$$m = \sum_{i=1}^{N} w_i \ \mathrm{erf}\Big(\sqrt{\frac{Nw_i}{2ar}} m\Big) \tag{36}$$

$$q = 1 - \frac{1}{NK} \sum_{i=1}^{N} \sqrt{\frac{2Nw_i}{\pi a r}} \ \exp\Big(-\frac{Nw_i}{2ar} m^2\Big) \tag{37}$$

$$r = \frac{q}{(1 - K + Kq)^2}, \tag{38}$$

which are Eqs. (8-10) presented in the main text. Detailed explanations on these solutions are given in Fig 2 of the main text.

5 Dependence of the phase boundaries on degree exponent

As γ is decreased, the $T-a$ phase diagram in Fig 1 of the main text undergoes drastic changes. Especially, the R phase introduces into the region of the SG phase, but it also raises the boundary of the P phase to a high-temperature region as γ is decreased. As γ approach 2.0, it is shown that the region of R phase becomes broader whereas the SG phase shrinks and eventually disappears. Such changes of the phase diagram in the $T-a$ space can be obtained by the leading term in the expansion of the right hand side of Eq. (30) with $m=0$, i.e., $q \simeq arK^2\mathbf{T}_2X$ with $X \equiv N\sum_{i=1}^{N} w_i^2$, under the condition $N^{1/(\gamma-1)} \ll 1$. From this, we obtain the glass transition temperature to be $T_g \simeq 1/K \tanh^{-1}(1/K(1+\sqrt{aX}))$. For the ER network ($\gamma \to \infty$) with $K=5$ and $a=0$, we obtain $T_g \simeq 1.013$ as shown in Fig 1(a) of the main paper. However, as $\gamma \to 2$, the condition $N^{1/(\gamma-1)} \ll 1$ cannot be fulfilled as $N \to \infty$, thus there exists no phase boundary between the P and SG phases and the R phase intrudes into the region between the two phases.

6 Dependence of the error rate on degree exponent

Fig 2 of the main text shows the error rate $n_e \equiv (1-m)/2$ as a function of storage rate a, obtained by Eqs. (36-38) for various γ values at zero temperature. As shown in Eq.(36), m has a γ dependence in terms of w_i. For the case $\gamma \to \infty$, Nw_i becomes unity and Eq.(36) reduces to $m = \mathrm{erf}(m/\sqrt{2ar})$, by which m suddenly becomes zero when a is larger than a specific value called the critical storage capacity $a_c = a_c(\gamma)$. For the case $\gamma \to 2+\epsilon$ ($\epsilon \ll 1$), however, $Nw_i \sim N^{1/(1+\epsilon)} i^{-1/(1+\epsilon)} \gg 1$ as $N \to \infty$, by which m becomes nonzero even for sufficiently large value of a and it is thus impossible to define the critical storage capacity a_c. Such a γ-dependence of m determines the behavior of the error rate n_e, as shown in Fig 2 of the main text.

7 Dependence of the phase diagrams on size N

We remark that even though we obtained analytic formulae of Sec. III in the thermodynamic limit, the phase diagrams (Figs 1 and 2 of the main text) were obtained in finite systems. We find that when γ is sufficiently large, Np_i is not so sensitive to N that the result of the ER case does not depend on N seriously, as shown in S3 Fig (a). However, when γ approaches 2.0, the R-phase region becomes wider as N is increased, as shown in S3 Fig (b).

8 Comparison between the Chung-Lu model and the static model

We show the phase diagram in the space $(T-a)$ and the error rate $n_e = (1-m)/2$ as a function of storage rate a for the static model. First, S4 Figs (a) and (b) show the phase diagrams for the static model with (a) $\gamma = 2.35$ and (b) $\gamma = 2.01$, which correspond to the Figs 1(e) and (f) of the main paper for the CL model. These phase diagrams for the static model are similar qualitatively to those for the CL model, but

the transitions temperature between P and R phases obtained for the static model is somewhat larger than the one for the CL model.

S5 Fig is the plot of the error rate $n_e \equiv (1-m)/2$ vs storage rate a at zero temperature for the two different γ values for the static model. This figure corresponds to Fig 2(b) of the main paper for the CL model. Whereas the SG phase of the CL model remains on the axis $T = 0$ when $\gamma \simeq 2.04$, as shown in Fig 2(b) of the main paper, the SG phase of the static model given in S4 Fig remains on the axis $T = 0$ when $\gamma \simeq 2.35$.

S6 Fig (a) and (b) show the comparison of the error rate for the two different models, the CL and the static models as a function of storage rate a for different degree exponent values (a) $\gamma = 2.01$ and (b) 2.04 at $T = 0$. These figures enable us to see qualitatively how the error rate depends on the degree-degree correlation. We recall that the disassortative degree-degree correlation is present for the static model in the region $2 < \gamma < 3$, but absent for the CL model. One can see that the degree-degree correlation reduces somewhat the error rate. Accordingly one may infer that the error rate of realistic brain networks, in which the degree-degree correlation is present, should be smaller than those obtained from the CL model analytically.

9 Hopfield model simulations on real neural networks

We check the error rate n_e of the Hopfield model on several real neural networks. For this purpose, we chose three real neural networks: the networks for Macaque monkey 1 (fve30; $N = 30$, $L = 311$) [17], Macaque monkey 2 (macaque47; $N = 47$, $L = 505$) [18] and cat (CIJctx; $N = 52$, $L = 818$) [19]. Those networks are constructed based on the connectivity network data sets [20]. The adjacency matrices of the three networks are given in the supplementary file(S1 Data Set). For the cat network, all links with nonzero elements (1, 2 and 3) of the adjacency matrix were regarded as connected.

Our simulations are performed following the random updating method of the Hopfield model on those real neural networks, which is the same as the method already used in Sec. 1(Hopfield model simulation on a random network). Here, the error rate $n_e(\equiv (1-m)/2)$ is calculated using the formula $m = (1/N)\sum_i^N \xi_i^1 S_i(t \to \infty)$ with $\mu = 1$. We perform 10^4 different runs with 10^4 different ensemble of $\{\xi_i^\mu\}$, and then obtain the error rate n_e over those ensemble.

S7 Fig shows the degree distributions (left column) and the error rates (right column) of those three different real neural networks. The degree distributions of three networks are heavy-tailed, but the degree exponents cannot be determined precisely due to the small system sizes of those networks. To compare the error rates obtained from the simulations with the ones from the theory, we draw the error rates obtained from both methods together in the right column of S7 Fig. Indeed, with appropriate weight $w_i(\gamma)$ near $\gamma = 2.0$, the two error rates behave similarly to each other as a function of the storage rate a.

References

1. Amit DJ. Modeling brain function: The world of attractor neural networks. Cambridge University Press; 1989.

2. Amit DJ, Gutfreund H, Sompolinsky H. Storing infinite numbers of patterns in a spin-glass model of neural networks. Phys Rev Lett. 1985 Sep;55(14):1530. doi:10.1103/PhysRevLett.55.1530.

3. Amit DJ, Gutfreund H, Sompolinsky H. Statistical mechanics of neural networks near saturation. Ann Phys (NY). 1987 Jan;173(1):30–67. doi:10.1016/0003-4916(87)90092-3.

4. Mézard M, Parisi G, Virasoro MA. Spin glass theory and beyond. World Scientific; 1987.

5. Nishimori H. Statistical physics of spin glasses and information processing: an introduction. Oxford University Press; 2001.

6. Viana L, Bray AJ. Phase diagrams for dilute spin glasses. J Phys C: Solid State Phys. 1985 Oct;18(15):3037–3051. doi:10.1088/0022-3719/18/15/013.

7. Kanter I, Sompolinsky H. Mean-field theory of spin-glasses with finite coordination number. Phys Rev Lett. 1987 Jan;58(2):164. doi:10.1103/PhysRevLett.58.164.

8. Mézard M, Parisi G. Mean-field theory of randomly frustrated systems with finite connectivity. EPL (Europhys Lett). 1987 Feb;3(10):1067–1074. doi:10.1209/0295-5075/3/10/002.

9. Mottishaw P, De Dominicis C. On the stability of randomly frustrated systems with finite connectivity. J Phys A: Math Gen. 1987 Jan;20(6):L375–L379. doi:10.1088/0305-4470/20/6/007.

10. Wong K, Sherrington D. Intensively connected spin glasses: towards a replica-symmetry-breaking solution of the ground state. J Phys A: Math Gen. 1988 Jan;21(8):L459–L466. doi:10.1088/0305-4470/21/8/006.

11. Monasson R. Optimization problems and replica symmetry breaking in finite connectivity spin glasses. J Phys A: Math Gen. 1998 Jul;31(2):513–529. doi:10.1088/0305-4470/31/2/012.

12. Kim DH, Rodgers G, Kahng B, Kim D. Spin-glass phase transition on scale-free networks. Phys Rev E. 2005 May;71(5):056115. doi:10.1103/PhysRevE.71.056115.

13. Kim DH. Inverse transitions in a spin-glass model on a scale-free network. Phys Rev E. 2014 Feb;89(2):022803. doi:10.1103/PhysRevE.89.022803.

14. Stratonovich RL. On a Method of Calculating Quantum Distribution Functions. Sov. Phys. Doklady. 1958;2:416.

15. Hubbard J. Calculation of Partition Functions. Phys. Rev. Lett. 1959;3(2):77–78. doi:10.1103/PhysRevLett.3.77.

16. De Almeida J, Thouless DJ. Stability of the Sherrington-Kirkpatrick solution of a spin glass model. J Phys A: Math Gen. 1978;11(5):983–990. doi:10.1088/0305-4470/11/5/028.

17. Felleman DJ, Van Essen DC. Distributed hierarchical processing in the primate cerebral cortex. Cereb Cortex. 1991 Jan;1(1):1–47. doi:10.1093/cercor/1.1.1.

18. Honey CJ, Kötter R, Breakspear M, Sporns O. Network structure of cerebral cortex shapes functional connectivity on multiple time scales. Proc Natl Acad Sci USA. 2007 Apr;104(24):10240–10245. doi:10.1073/pnas.0701519104.

19. Scannell J, Burns G, Hilgetag C, O'Neil M, Young MP. The connectional organization of the cortico-thalamic system of the cat. Cereb Cortex. 1999 Apr;9(3):277–299. doi:10.1093/cercor/9.3.277.

20. Rubinov M, Kötter R, Hagmann P, Sporns O. Brain connectivity toolbox: a collection of complex network measurements and brain connectivity datasets. NeuroImage. 2009 Aug;47:S169. doi:10.1016/S1053-8119(09)71822-1. (See https://sites.google.com/site/bctnet/datasets)
